# Genome-Wide Association Study Identifies Eight Novel Loci for Susceptibility of Scrub Typhus and Highlights Immune-Related Signaling Pathways in Its Pathogenesis

**DOI:** 10.3390/cells10030570

**Published:** 2021-03-05

**Authors:** Yong-Chan Kim, Soriul Kim, Hee-Kwon Kim, Yi Lee, Chol Shin, Chang-Seop Lee, Byung-Hoon Jeong

**Affiliations:** 1 Korea Zoonosis Research Institute, Jeonbuk National University, Iksan, Jeonbuk 54531, Korea; kych@jbnu.ac.kr; 2 Department of Bioactive Material Sciences, Jeonbuk National University, Jeonju, Jeonbuk 54896, Korea; 3 Institute for Human Genomic Study, College of Medicine, Korea University, Seoul 02841, Korea; soriul@korea.ac.kr (S.K.); chol-shin@korea.ac.kr (C.S.); 4 Molecular Imaging & Therapeutic Medicine Research Center, Department of Nuclear Medicine, Biomedical Research Institute, Jeonbuk National University Medical School and Hospital, Jeonju, Jeonbuk 54907, Korea; hkkim717@jbnu.ac.kr; 5 Department of Industrial Plant Science & Technology, Chungbuk National University, Chungju, Chungbuk 28644, Korea; leeyi22@cbnu.ac.kr; 6 Department of Internal Medicine, Division of Pulmonary Sleep and Critical Care Medicine, Korea University Ansan Hospital, Ansan 15355, Korea; 7 Department of Internal Medicine, Research Institute of Clinical Medicine, Jeonbuk National University Medical School, Jeonju, Jeonbuk 54907, Korea; 8 Biomedical Research Institute, Jeonbuk National University Hospital, Jeonju, Jeonbuk 54907, Korea

**Keywords:** scrub typhus, *Orientia tsutsugamushi*, genome-wide association study, GWAS, MIST, PANTHER, signal pathway

## Abstract

Scrub typhus is a fatal zoonotic disease caused by *Orientia tsutsugamushi*. This disease is accompanied by systemic vasculitis, lymphadenopathy, headache, myalgia, and eschar. In recent studies, a novel strain that is resistant to current medical treatment was identified in Thailand. Thus, the development of new specific drugs for scrub typhus is needed. However, the exact molecular mechanism governing the progression of scrub typhus has not been fully elucidated. To understand disease-related genetic factors and mechanisms associated with the progression of scrub typhus, we performed a genome-wide association study (GWAS) in scrub typhus-infected patients and found a scrub typhus-related signaling pathway by molecular interaction search tool (MIST) and PANTHER. We identified eight potent scrub typhus-related single nucleotide polymorphisms (SNPs) located on the *PRMT6, PLGLB2, DTWD2, BATF, JDP2, ONECUT1, WDR72, KLK, MAP3K7*, and *TGFBR2* genes using a GWAS. We also identified 224 genes by analyzing protein-protein interactions among candidate genes of scrub typhus and identified 15 signaling pathways associated with over 10 genes by classifying these genes according to signaling pathways. The signaling pathway with the largest number of associated genes was the gonadotropin-releasing hormone receptor pathway, followed by the TGF-beta signaling pathway and the apoptosis signaling pathway. To the best of our knowledge, this report describes the first GWAS in scrub typhus.

## 1. Introduction

Scrub typhus is a zoonotic disease caused by *Orientia tsutsugamushi*, a Gram-negative family of Rickettsiaceae bacteria spread by chigger mites [1,2]. One billion people have been threatened by this disease globally, and more than one million new cases have been reported every year, mostly in the Asia-Pacific area [3,4]. Scrub typhus is an acute febrile disease accompanied by systemic vasculitis, lymphadenopathy, headache, myalgia, and eschar [5,6,7]. Since scrub typhus was first discovered in the 1930s in Japan, the extent of infection has been widening as global warming and globalization progress [2]. The fatality rates vary widely around a median mortality of 1.4% for treated patients and 6.0% for untreated patients [8]. The first treatment of scrub typhus is antibiotics, including doxycycline or azithromycin, but chloramphenicol is an alternative [9]. A novel strain that is resistant to doxycycline and chloramphenicol has been identified in Thailand; however, there is some controversy [10,11,12,13]. Thus, the development of new specific drugs for scrub typhus is needed. Moreover, the exact molecular mechanism of scrub typhus progression has not been fully elucidated to date. 

Because scrub typhus is a Rickettsia disease, it is not surprising that several studies have noted Rickettsia-related innate immune response proteins [14]. In this context, an important study using the candidate gene approach has been performed to identify the pathogenesis of scrub typhus associated with molecular mechanisms [15]. Single nucleotide polymorphism (SNP) of the heat shock protein 70 (*HSP* 70) gene showed an association with clinical outcomes in intensive care unit patients with scrub typhus-induced sepsis [15]. In addition, the nonsynonymous SNP Asp299Gly of the toll-like receptor (*TLR*) 4 gene was significantly associated with susceptibility to scrub typhus [13]. Although association studies may help to characterize the relationship between disease and host genes [16,17,18,19], the investigation was only performed in immune-related genes, and scrub typhus-related specific molecular pathways have been elusive to date. In recent studies, genome-wide association study (GWAS), which is a high-throughput convenient genetic method to generate or reveal disease-related molecular mechanisms based on genome-wide compared data between case and control populations using highly integrated SNP chips, has been widely used [20,21,22]. 

Thus, in the present study, we performed a GWAS in scrub typhus-infected patients to develop novel candidate genes that contribute to the pathogenesis of scrub typhus. In addition, we analyzed linkage disequilibrium (LD) to evaluate whether scrub typhus-related SNPs were in strong genetic linkage. Furthermore, we used a molecular interaction search tool (MIST, http://fgrtools.hms.harvard.edu/MIST/) to find protein-protein interaction partners of candidate genes of scrub typhus [23]. Finally, we analyzed the signaling pathway and gene function of scrub typhus-related molecules by PANTHER (http://www.pantherdb.org/tools/csnpScoreForm.jsp) [24].

## 2. Materials and Methods

### 2.1. Ethical Statement

All samples were obtained with informed consent under institutional review board-approved protocols. All procedures performed in the present study were approved according to guidelines of the institutional review board of Jeonbuk National University Hospital and in accordance with the 1964 Helsinki declaration and its later amendments or comparable ethical standards (approval number: IRB No. CUH 2016-09-005). All the samples and related data were anonymized prior to investigation.

### 2.2. Subjects

Blood samples of 74 Korean patients with laboratory-confirmed scrub typhus were provided by Jeonbuk National University Hospital. The diagnosis of patients with scrub typhus was confirmed by an increase in the indirect immunofluorescence assay (IFA) IgM titer ≥ 1:160 against *O. tsutsugamushi* or a ≥ 4-fold increase in the IFA titer *O. tsutsugamushi*, or when a positive reaction was observed in a nested polymerase chain reaction (PCR) targeting the 56 kDa gene of *O. tsutsugamushi*. All patients showed mild symptoms and were diagnosed at an early stage. Genomic DNA was purified from 200 μL of blood using a Blood Genomic DNA Isolation kit (Qiagen, Valencia, CA, USA) following the manufacturer’s instructions. The GWAS data of 74 healthy Koreans were provided by Korea University. The exclusion criteria of healthy Koreans were asthma, chronic obstructive pulmonary disease, hepatitis, tuberculosis, head trauma, urinary tract infection, and cancer. The 74 healthy Koreans have no history of scrub typhus infection. 

### 2.3. GWAS 

Genomic DNA with 1.8–2.0 values of A260/A280 was used for genotyping. GWAS was performed by the Axiom™ Asia Precision Medicine Research Array (AMPRA) using a 500K SNP Chip (Thermo Fisher, Waltham, MA, USA). Genotyping was carried out using a Gaussian likelihood model. Quality control (QC) of genotyping data was performed, and SNPs with the following conditions were excluded: Hardy-Weinberg equilibrium (HWE) test, *p* value < 1 × 10^−4^; call rate <95%; minor allele frequency (MAF) <1%. Statistical analysis for identifying candidate SNPs was performed by Plink v1.90 (https://www.cog-genomics.org/plink2) and performed multivariate logistic regression models, including age and gender as covariates. Candidate SNPs with *p* < 0.0001 were evaluated and excluded by Cluster QC. The plots were visualized by R v4.0.4 (https://www.r-project.org/) and LocusZoom v0.12 (http://locuszoom.org/). Candidate SNPs with *p* value < 1 × 10^−3^ were analyzed by linkage disequilibrium (LD) test, and LD block was visualized by Haploview v4.2 (Broad Institute, Cambridge, MA, USA). Statistical analyses were performed using SAS v9.4 (SAS Institute Inc., Cary, NC, USA). Statistical significance using *p* value was calculated with a two-tailed Student’s *t* test and χ^2^ test.

### 2.4. In Silico Analysis of Scrub Typhus-Related Candidate Genes and Signaling Pathways

The biological interaction data were collected by the MIST database v5.0 (http://fgrtools.hms.harvard.edu/MIST/) from model systems of several species, as well as humans. MIST can identify interacting partners based on protein–protein and genetic interaction data from the species of interest, as well as inferred interactions and visualize a corresponding network. PANTHER v16.0 (http://www.pantherdb.org/tools/csnpScoreForm.jsp) is a comprehensive web-based platform for analyzing large-scale genome and gene function. PANTHER provides query gene functions and analyzes large-scale experimental data with a number of statistical tests.

## 3. Results

### 3.1. Subjects

A total of 148 individuals were included in the GWAS analysis. Detailed information on the study population is described in Table 1. A total of 74 scrub typhus-diagnosed patients were composed of 43 females and 31 males. A total of 74 healthy individuals were composed of 42 females and 32 males. The two groups showed a similar distribution of sex (*p* = 0.87). The mean age of diagnosis of scrub typhus patients was 64.4 ± 7.6 years, and the mean age of healthy individuals at sample collection was 64.4 ± 7.6 years. The mean ages between the two groups showed a very similar distribution (*p* = 0.93).

### 3.2. GWAS for Scrub Typhus

GWAS was performed in QC and passed 477,093 SNPs using the Axiom™ AMPRA 500K SNP. A total of 1485 SNPs with *p* < 1 × 10^−4^ values were refined by cluster QC, and 39 SNPs passed QC (Table 2).

**Figure 1:**
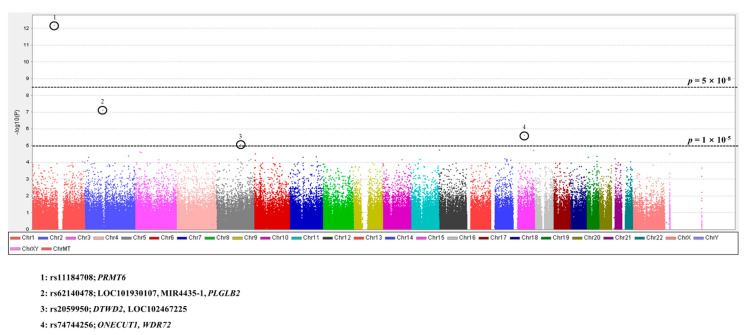
Manhattan plot of the strength of association between scrub typhus-infected patients and healthy age- and sex-matched controls. *Y* axis: −log10(*p*) values; *X* axis: chromosomal position. Dotted lines indicate two thresholds of potent association with *p* value = 1 × 10^−5^ and *p* value = 5 × 10^−8^.

Detailed information on candidate SNPs of scrub typhus with *p* < 1 × 10^−4^ is described in Supplementary Table S1. Notably, four SNPs showed potent associations with *p* < 1 × 10^−5^. The top SNP related to susceptibility of scrub typhus was the rs11184708 SNP, which is located upstream of the protein arginine methyltransferase 6 (*PRMT6*) gene with a *p* = 6.447 × 10^−13^ value. The second SNP was the rs62140478 SNP, which is located upstream of the plasminogen-like B2 (*PLGLB2*) gene with a *p* = 7.404 × 10^−8^ value. Rs2059950 of the DTW domain containing 2 (*DTWD2*) gene and rs74744256 of the one cut homeobox 1 (*ONECUT1*) and WD repeat domain 72 (*WDR72*) gene also showed a significant association of scrub typhus with *p* < 1 × 10^−5^. A genomic overview of the association of susceptibility of scrub typhus was visualized by a Manhattan plot (Figure 1).

**Figure 2:**
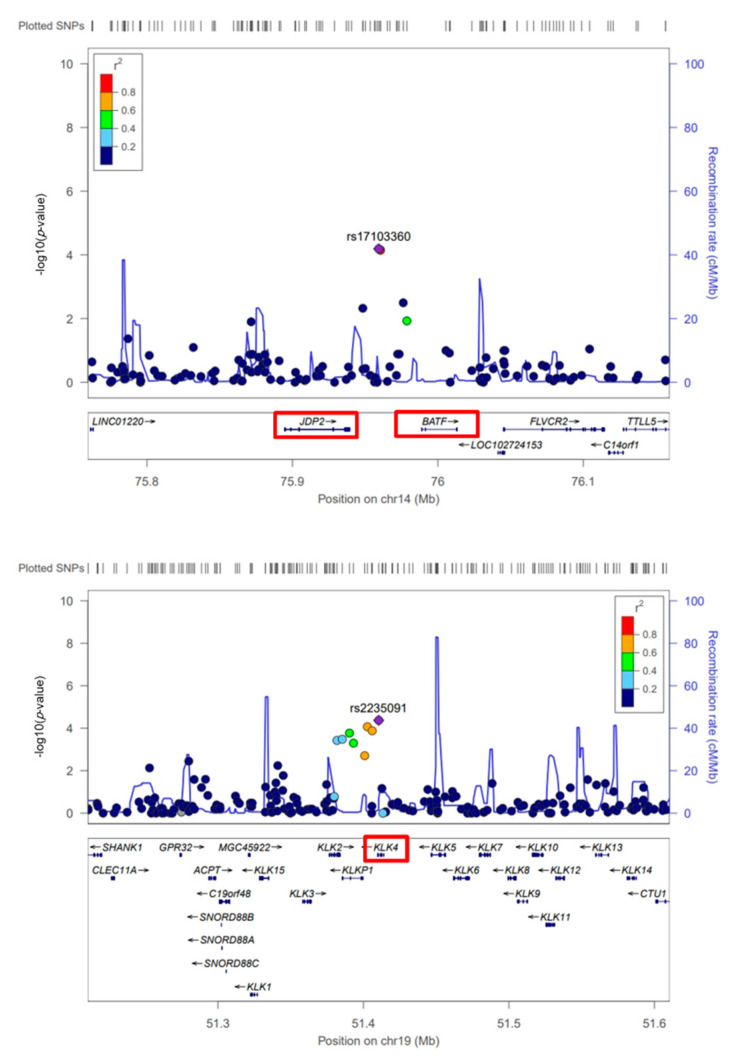
Illustration of association signals from strong linkage disequilibrium (LD) showed loci (top: *JDP2* and *BATF* gene loci on chromosome 14; bottom: *KLK4* gene locus on chromosome 19). Red boxes indicate the nearest genes with associated single nucleotide polymorphisms (SNPs). The diagram was visualized by LocusZoom (http://locuszoom.org/). AX-1282496: rs17103360; AX-12824299: rs7155418; AX-86334747: rs7155603; AX-40466275: rs198977; AX-11665883: rs8103659; AX-32775669: rs198956; AX-96079990: rs1354774; AX-11280320: rs1654513; AX-11663123: rs8103659; AX-40466329: rs2235091. The color indicates values of LD.

Among candidate SNPs with *p* < 1 × 10^−4^, rs17103360, rs7155418, and rs7155603 SNPs were located near the basic leucine zipper ATF-like transcription factor (*BATF*) and Jun dimerization protein 2 (*JDP2*) genes in chromosome 14. In addition, rs1654513 and rs2235091 SNPs were located near the kallikrein-related peptidase 4 (*KLK4*) gene in chromosome 19 (Supplementary Table S1). Regional plots were drawn by LocusZoom (Figure 2).

**Figure 3:**
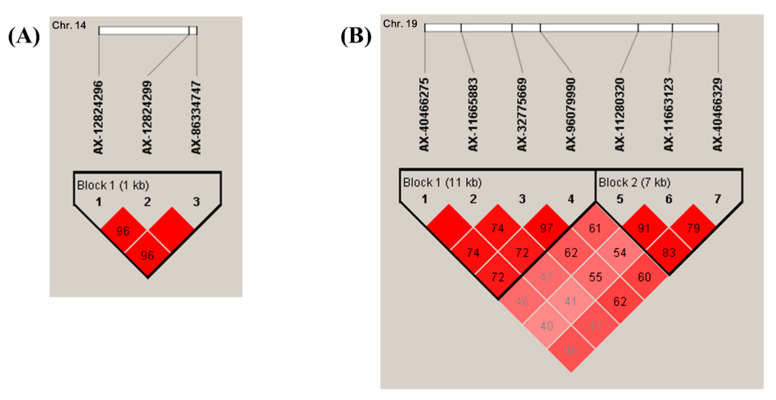
Linkage disequilibrium (LD) structure identified in chromosomes 14 and 19. (**A**) LD block located on the adjacent region of the *JDP2* and *BATF* genes on chromosome 14. (**B**) LD blocks located on the adjacent region of the *KLK4* gene on chromosome 19. The color gradient indicates values of LD.

SNPs with *p* < 1 × 10^−3^ that showed strong genetic linkage with regionally clustered SNPs with *p* < 1 × 10^−4^ are listed in Supplementary Table S2. In addition, we analyzed strong LD blocks among these SNPs (Figure 3). Notably, three strong LD blocks were identified, including one LD block in chromosome 14 and two LD blocks in chromosome 19. The strong LD block in chromosome 14 was composed of three SNPs, including AX-1282496 (rs17103360), AX-12824299 (rs7155418), and AX-86334747 (rs7155603), and it was stretched approximately 1 kb (Supplementary Table S2, Figure 3A). Two strong LD blocks were found on chromosome 19. Of these two blocks, LD block 1 was composed of four SNPs, including AX-40466275 (rs198977), AX-11665883 (rs8103659), AX-32775669 (rs198956), and AX-96079990 (rs1354774), and was stretched approximately 11 kb. LD block 2 was composed of three SNPs, including AX-11280320 (rs1654513), AX-11663123 (rs8103659) and AX-40466329 (rs2235091), and was stretched to 7 kb (Supplementary Table S2, Figure 3B). Next, we analyzed the signaling pathways of 39 candidate genes of scrub typhus with a *p* value < 1 × 10^−4^ using PANTHER (Supplementary Table S3). Notably, the mitogen-activated protein kinase kinase kinase 7 (*MAP3K7*) gene and TGF-beta receptor type-2 (*TGFBR2*) gene belong to the TGF-beta signaling pathway.

### 3.3. In Silico Analysis of Scrub Typhus-Related Candidate Genes and Signaling Pathways

**Figure 4:**
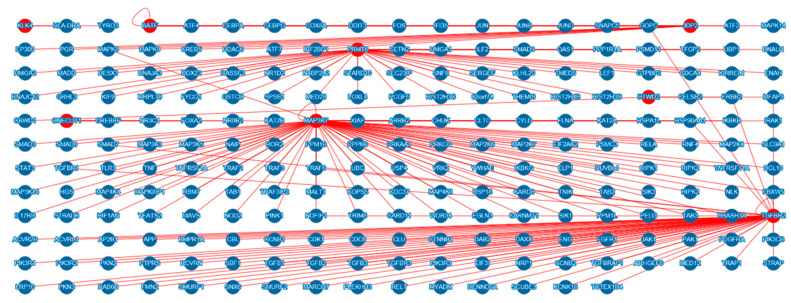
Protein-protein interaction genes with candidate genes of scrub typhus. Protein-protein interaction genes with candidate genes of scrub typhus were analyzed by MIST v5.0 (http://fgrtools.hms.harvard.edu/MIST/) using grid layout. Red circles indicate candidate genes of scrub typhus. Red lines indicate protein-protein interactions.

A total of eight candidate genes of scrub typhus, that is, *PRMT6, DTWD2, BATF, JDP2, ONCUT1*, *KLK4, MAP3K7*, and *TGFBR2*, with *p* value < 1 × 10^−5^, belonging to strong LD blocks or belonging to the same signal pathway, were analyzed by MIST to identify protein-protein interaction partners. A total of 224 genes predicted protein-protein interactions with candidate genes of scrub typhus were identified. The protein-protein interaction network of genes that interact with candidate genes of scrub typhus is visualized in Figure 4. We classified these genes that interact with candidate genes of scrub typhus according to the signaling pathway by PANTHER (Supplementary Table S4). A total of 62 signal pathways were identified. Among these signaling pathways, over 10 genes were associated with 15 signaling pathways, including angiogenesis, apoptosis signaling pathway, CCKR signaling map, EGF receptor signaling pathway, FGF signaling pathway, gonadotropin-releasing hormone receptor pathway, inflammation mediated by chemokine and cytokine signaling pathway, integrin signaling pathway, PDGF signaling pathway, Ras pathway, TGF-beta signaling pathway, Toll receptor signaling pathway, Wnt signaling pathway, and p53 pathway. Detailed information on signal pathway-related genes is shown in Supplementary Table S4. The signaling pathway with the largest number of associated genes was the gonadotropin-releasing hormone receptor pathway (31 genes) followed by the TGF-beta signaling pathway (25 genes) and the apoptosis signaling pathway (24 genes).

Finally, we investigated the immune-related function of genes that interact with candidate genes of scrub typhus based on protein-protein interactions. A total of five genes, including interleukin enhancer binding factor 2 (*ILF2*), 2’-5’-oligoadenylate synthetase 1 (*OAS1*), *BATF*, interferon-induced protein 35 (*IFI35*) and major histocompatibility complex, class II, DR alpha (*HLA-DRA*) genes, showed immune-related gene function. Detailed information on these genes is shown in Table 3.

## 4. Discussion

In the present study, we carried out a genome-wide scale association study in scrub typhus patients for the first time. We identified a total of eight SNPs located on adjacent regions of the *PRMT6, PLGLB2, DTWD2, BATF, JDP2, ONECUT1, WDR72, KLK, MAP3K7*, and *TGFBR2* genes, which had a significant association with susceptibility to scrub typhus (Supplementary Table S1). Rs11184708, rs62140478, rs2059950, and rs74744256 showed significant associations with *p* < 1 × 10^−5^. In addition, rs17103360 and rs2235091 showed a significant association with *p* < 1 × 10^−4^, and adjacent SNPs showed strong LD (Supplementary Table S2) and composed LD blocks located on chromosomes 14 and 19. Furthermore, rs6809058 and rs16883596 belonged to the same signaling pathway. Because 10 candidate genes of scrub typhus were multifunctional genes, the role of the pathogenesis of scrub typhus was not determined. Thus, we analyzed the protein-protein interactions of candidate genes of scrub typhus. A total of 224 genes that interact with candidate genes of scrub typhus have been identified based on protein-protein interactions (Figure 4). To identify the scrub typhus-related signaling pathway, we classified 224 genes according to the signaling pathway. We postulated that multiple genes involved in the signaling pathway would be more affected by the scrub typhus-related signaling pathway. We identified 15 signal pathways involved in over 10 genes that interact with candidate genes of scrub typhus based on protein-protein interactions (Supplementary Table S4). The signaling pathway with the largest number of associated genes was the gonadotropin-releasing hormone receptor pathway. Since epidemiological studies reported that the susceptibility of women to scrub typhus was higher than that of men, further research investigating the association between susceptibility according to sex and the gonadotropin-releasing hormone receptor pathway is warranted [25]. TLR, which had been shown to be associated with susceptibility to scrub typhus in a previous study, also reaffirmed its association with the signaling pathway of scrub typhus [15]. Several immune-related signaling pathways, including the TGF-beta signaling pathway, Toll receptor signaling pathway, T cell activation, inflammation mediated by chemokine and cytokine signaling pathways, showed an association with scrub typhus. Further research on the CCKR signaling map, EGF receptor signaling pathway, FGF signaling pathway, PDGF signaling pathway, Wnt signaling pathway, and p53 pathway is needed in the future. Previous studies reported that the surface antigen of *O. tsutsugamushi* activates the nuclear factor-kB and p38 mitogen-activated protein kinase pathway and that intracellular invasion of *O. tsutsugamushi* is mediated by integrin signaling [26]. These signaling pathways were also identified in the present study (Supplementary Table S4).

Finally, we examined the function of genes that interact with candidate genes of scrub typhus based on protein-protein interactions. Five genes, including *ILF2, OAS1, BATF, IFI35*, and *HLA-DRA* genes, showed apparent immune-related functions. The *BATF* gene located downstream of the *JDP2* gene and adjacent GWAS SNP markers showed a significant association and composed a strong LD block. In addition, since the *IFI35* gene is a downstream effector of these two genes, further investigation of the association of these genes with susceptibility to scrub typhus is highly desirable in the future. Furthermore, the *ILF2* and *OAS* genes were downstream of the top SNP, and the rs11184708 SNP located upstream of the *PRMT6* gene, which is related to susceptibility to scrub typhus. Further research investigating the relationship using overexpression and knockdown/out studies of these genes in cell culture and animal models of scrub typhus is warranted in the future.

However, since all scrub typhus patients showed mild symptoms and were diagnosed at an early stage, there may be bias in the data obtained. In addition, since the severity of infection ultimately could dictate the outcomes of the GWAS, including signal pathways and gene expression, further repetitive stratified GWAS analysis is highly desirable in patients showing various phenotypes, including the various degrees of symptoms and stages of infection.

## 5. Conclusions

In conclusion, in the present study, we performed GWAS in scrub typhus-infected patients. We identified eight potent scrub typhus-related SNPs located on the *PRMT6, PLGLB2, DTWD2, BATF, JDP2, ONECUT1, WDR72, KLK, MAP3K7*, and *TGFBR2* genes. We analyzed protein-protein interaction genes with candidate genes of scrub typhus and identified 224 genes. We classified these genes according to signaling pathways and identified 15 signaling pathways associated with over 10 genes. The signaling pathway with the largest number of associated genes was the gonadotropin-releasing hormone receptor pathway. To the best of our knowledge, this report is the first systemic GWAS analysis to identify novel susceptible genetic biomarkers for scrub typhus infection.

## Figures and Tables

**Table 1:** Summary of information of subjects in this study.

Characteristics		Case	Control
Number		74	74
Sex	Female	43	42
	Male	31	32
	*p* value		0.87
Mean age of diagnosis (years ± *STD)		64.4 ± 7.6	
Mean age at sample collection (years ± *STD)			64.4 ± 7.6
	*p* value		1.0

*STD: Standard deviation.

**Table 2:** Detailed information of single nucleotide polymorphisms (SNPs) analyzed in this study.

*p* Value *	Count	Cumulative
<1 × 10^−6^	2	2
<1 × 10^−5^	2	4
<1 × 10^−4^	35	39
<1 × 10^−3^	791	830
<0.01	4219	5049
<0.05	17,303	22,352
NS**	454,741	477,093

* *p* value indicates genetic difference of allele frequencies between case and control populations. ** NS: Not significant.

**Table 3:** Immune-related genes that interact with candidate genes of scrub typhus based on protein-protein interaction.

Gene	Gene Full Name	Function	Related Candidate Gene of Scrub Typhus
*ILF2*	Interleukin enhancer binding factor 2	Transcription factor required for T-cell expression of the interleukin 2 gene	*PRMT6*
*OAS1*	2’-5’-oligoadenylate synthetase 1	Interferon-induced, dsRNA-activated antiviral enzyme which plays a critical role in cellular innate antiviral response	*PRMT6*
*BATF*	Basic leucine zipper ATF-like transcription factor	AP-1 family transcription factor that controls the differentiation of lineage-specific cells in the immune system	*−*
*IFI35*	Interferon induced protein 35	*IFI35* involved in interferon gamma signaling and innate immune system	*BATF, JDP2*
*HLA-DRA*	Major histocompatibility complex, class II, DR alpha	*HLA-DRA* plays a central role in the immune system by presenting peptides derived from extracellular proteins	*KLK4*

## Data Availability

The data that support the findings of this study are available from the corresponding author upon reasonable request.

## Supplementary Materials

The following are available online at https://www.mdpi.com/2073-4409/10/3/570/s1, Table S1: Detailed information on scrub typhus-related candidate SNPs with a *p* value < 1 × 10^−4^; Table S2: Linkage disequilibrium (LD) of scrub typhus candidate SNPs with a *p* value < 1 × 10^−3^; Table S3: Signaling pathway of scrub typhus-related candidate SNPs with *p* value < 1 × 10^−4^; Table S4: Signaling pathways of genes that interact with candidate genes of scrub typhus based on protein-protein interactions.

## Abbreviations

GWAS	Genome-wide association study
SNP	Single nucleotide polymorphism
HSP70	Heat Shock Protein 70
TLR4	Toll-like receptor 4
PRMT6	Protein arginine methyltransferase 6
ONECUT1	One cut homeobox 1
WDR72	WD repeat domain 72
BATF	Basic leucine zipper ATF-like transcription factor
JDP2	Jun dimerization protein 2
KLK4	Kallikrein-related peptidase 4
MAP3K7	Mitogen-activated protein kinase kinase kinase 7
TGFBR2	TGF-beta receptor type-2
ILF2	Interleukin enhancer binding factor 2
OAS1	2’-5’-oligoadenylate synthetase 1
IFI35	Interferon-induced protein 35
HLA-DRA	Major histocompatibility complex, class II, DR alpha
