# Supplementary material for: Genome-Wide Association Study Identifies Eight Novel Loci for Susceptibility of Scrub Typhus and Highlights Immune-Related Signaling Pathways in Its Pathogenesis
Source: Cells. 2021 Mar 5;10(3):570. doi: 10.3390/cells10030570 (PMC7999653; doi:10.3390/cells10030570)
Supplement: Supplementary file 1 [file cells-10-00570-s001.pdf]

cells MDPI

Supplementary Table S1. Detailed information on scrub typhus-related candidate SNPs with a p value < 1 × 10^{-4}.

SNP	Gene	Chr*	Region	p value	Odd Ratios	Minor Allele	Major Allele
rs11184708	*PRMT6*	1	upstream	6.447× 10^{-13}	6.149	T	A
rs10802595	*RYR2*	1	intron	0.00008738	2.593	A	G
rs401974	*LINC00276,LOC100506474*	2	downstream, intron, upstream	0.0000769	0.3921	T	C
rs1445126	*MIR4757,NT5C1B*	2	upstream	0.00004819	3.18	A	G
rs62140478	*LOC101930107,MIR4435-1,PLGLB 2*	2	downstream, upstream	7.404 × 10^{-8}	9.708	T	C
rs35890165	*CPS1,ERBB4*	2	downstream	0.00003952	0.373	A	G
rs34599430	*ZNF385D,ZNF385D-AS2*	3	intron, upstream	0.00002317	2.767	G	A
rs6809058	*RBMS3,TGFBR2*	3	downstream, upstream	0.00002507	3.094	G	A
rs3773683	*SIDT1*	3	intron	0.00006635	0.3543	C	T
rs11727383	*CRMP1,EVC*	4	intron	0.0000803	0.3459	A	G
rs17338338	*NUDT12,RAB9BP1*	5	upstream	0.00006987	0.3746	G	A
rs2059950	*DTWD2,LOC102467225*	5	downstream	0.000008739	2.911	G	A
rs72663337	*DTWD2,LOC102467225*	5	downstream	0.00009856	2.566	T	C
rs6882516	*LSM11*	5	UTR-3	0.00007553	2.968	A	C
rs76949230	*TENM2*	5	intron	0.00006305	3.048	G	C
rs3804468	*LY86,LY86-AS1*	6	intron	0.00003155	0.202	C	T
rs3778337	*DSP*	6	exon,intron	0.00007311	0.3897	G	A
rs16883596	*MAP3K7,MIR4643*	6	upstream	0.00005186	4.274	A	G
rs35144103	*CCT6P3,ZNF92*	7	downstream, upstream	0.00004885	3.422	A	G
rs13244090	*LOC407835,TPI1P2*	7	downstream, upstream	0.00004505	2.638	G	A
rs17167553	*LRGUK*	7	missense	0.00008788	2.75	T	G
rs6583826	*IDE,KIF11*	10	upstream	0.00006894	0.2846	G	A
rs10769111	*LOC221122,PRDM11*	11	downstream, upstream	0.0000619	0.3816	T	G
rs10848921	*EFCAB4B*	12	intron	0.00001737	0.18	T	C
rs17836777	*TMEM229B*	14	intron	0.0000604	2.939	C	T
**rs17103360**	***BATF, JDP2***	**14**	**downstream, upstream**	**0.0000646**	**2.797**	**A**	**G**
**rs7155418**	***BATF, JDP2***	**14**	**downstream, upstream**	**0.0000709**	**2.762**	**G**	**A**
**rs7155603**	***BATF, JDP2***	**14**	**downstream, upstream**	**0.0000709**	**2.762**	**G**	**A**
rs2270324	*VASH1*	14	intron	0.00008391	0.2632	G	A
rs1187740	*SNHG10,SYNE3*	14	downstream, upstream	0.00007363	2.566	G	A
rs74744256	*ONECUT1,WDR72*	15	downstream, upstream	0.000002395	6.959	T	C
rs11247262	*FAM169B,LOC101927332*	15	downstream, upstream	0.00001947	0.3415	G	A
rs10416379	*OR7G1,OR7G2*	19	downstream, upstream	0.00004886	0.3704	T	C
rs386976	*ZNF682,ZNF93*	19	downstream, intron	0.000011	0.309	T	C
**rs1654513**	***KLK4,KLKP1***	**19**	**downstream, upstream**	**0.00008553**	**3.379**	**T**	**C**
**rs2235091**	***KLK4***	**19**	**intron**	**0.00004254**	**3.846**	**G**	**A**
rs446561	*LOC388813,NRIP1*	21	downstream, upstream	0.00006095	2.717	T	C
rs134897	*LINC01315,OGFRP1,TCF20*	22	downstream, intron	0.00009087	0.3745	T	C
rs142305985	*PPP2R3B,SHOX*	25	upstream	0.00003155	0.202	T	C

* Chr: Chromosome. Shaded blocks indicate scrub typhus-related candidate SNPs with a p value < 1 × 10^{-5}. Bold text indicates SNPs detected in the nearest region of the same genes.

Supplementary Table S2. Linkage disequilibrium (LD) of scrub typhus candidate SNPs with a p value < 1 × 10^{-3}.

Chr*	SNP A**	SNP B	r^2 Value***	Nearest Gene
14	rs17103360	rs17103360	1	*BATF, JDP2*
14	rs17103360	rs7155418	0.968458	*BATF, JDP2*
14	rs17103360	rs7155603	0.968458	*BATF, JDP2*
14	rs7155418	rs17103360	0.968458	*BATF, JDP2*
14	rs7155418	rs7155418	1	*BATF, JDP2*
14	rs7155418	rs7155603	1	*BATF, JDP2*
14	rs7155603	rs17103360	0.968458	*BATF, JDP2*
14	rs7155603	rs7155418	1	*BATF, JDP2*
14	rs7155603	rs7155603	1	*BATF, JDP2*
19	rs198977	rs198977	1	*KLK4*
19	rs198977	rs8103659	1	*KLK4*
19	rs198977	rs198956	0.760827	*KLK4*
19	rs198977	rs1354774	0.743057	*KLK4*
19	rs198977	rs1654513	0.493349	*KLK4*
19	rs198977	rs806019	0.433832	*KLK4*
19	rs198977	rs2235091	0.499187	*KLK4*
19	rs8103659	rs198977	1	*KLK4*
19	rs8103659	rs8103659	1	*KLK4*
19	rs8103659	rs198956	0.7588	*KLK4*
19	rs8103659	rs1354774	0.740866	*KLK4*
19	rs8103659	rs1654513	0.502075	*KLK4*
19	rs8103659	rs806019	0.441811	*KLK4*
19	rs8103659	rs2235091	0.507512	*KLK4*
19	rs198956	rs198977	0.760827	*KLK4*
19	rs198956	rs8103659	0.7588	*KLK4*
19	rs198956	rs198956	1	*KLK4*
19	rs198956	rs1354774	0.98009	*KLK4*
19	rs198956	rs1654513	0.634147	*KLK4*
19	rs198956	rs806019	0.566183	*KLK4*
19	rs198956	rs2235091	0.629552	*KLK4*
19	rs1354774	rs198977	0.743057	*KLK4*
19	rs1354774	rs8103659	0.740866	*KLK4*
19	rs1354774	rs198956	0.98009	*KLK4*
19	rs1354774	rs1354774	1	*KLK4*
19	rs1354774	rs1654513	0.590931	*KLK4*
19	rs1354774	rs806019	0.525722	*KLK4*
19	rs1354774	rs2235091	0.587585	*KLK4*
19	rs1654513	rs198977	0.493349	*KLK4*
19	rs1654513	rs8103659	0.502075	*KLK4*
19	rs1654513	rs198956	0.634147	*KLK4*
19	rs1654513	rs1354774	0.590931	*KLK4*
19	rs1654513	rs1654513	1	*KLK4*
19	rs1654513	rs806019	0.914265	*KLK4*
19	rs1654513	rs2235091	0.831862	*KLK4*
19	rs8103659	rs198977	0.433832	*KLK4*
19	rs8103659	rs8103659	0.441811	*KLK4*
19	rs8103659	rs198956	0.566183	*KLK4*
19	rs8103659	rs1354774	0.525722	*KLK4*
19	rs8103659	rs1654513	0.914265	*KLK4*

19	rs8103659	rs806019	1	*KLK4*
19	rs8103659	rs2235091	0.794065	*KLK4*
19	rs2235091	rs198977	0.499187	*KLK4*
19	rs2235091	rs8103659	0.507512	*KLK4*
19	rs2235091	rs198956	0.629552	*KLK4*
19	rs2235091	rs1354774	0.587585	*KLK4*
19	rs2235091	rs1654513	0.831862	*KLK4*
19	rs2235091	rs806019	0.794065	*KLK4*
19	rs2235091	rs2235091	1	*KLK4*

*Chr: Chromosome; **SNP A is candidate SNPs of scrub typhus with a p value < 1×10^{-4}; ***r^2 value: LD values between SNP A and SNP B.

Supplementary Table S3. Signaling pathway of scrub typhus-related candidate SNPs with p value < 1×10^{-4}.

Signal Pathway	Gene	Gene Full Name
Alzheimer disease-presenilin pathway	*ERBB4*	Receptor tyrosine-protein kinase erbB-4
Apoptosis signaling pathway	*JDP2*	Jun dimerization protein 2
Arginine biosynthesis	*CPS1*	Carbamoyl-phosphate synthase
Axon guidance mediated by semaphorins	*CRMP1*	Dihydropyrimidinase-related protein 1
Beta1 adrenergic receptor signaling pathway	*RYR2*	Ryanodine receptor 2
Beta2 adrenergic receptor signaling pathway	*RYR2*	Ryanodine receptor 2
CCKR signaling map	*RYR2*	Ryanodine receptor 2
Cadherin signaling pathway	*ERBB4*	Receptor tyrosine-protein kinase erbB-4
De novo pyrimidine ribonucleotides biosythesis	*CPS1*	Carbamoyl-phosphate synthase
EGF receptor signaling pathway	*ERBB4*	Receptor tyrosine-protein kinase erbB-4
Gonadotropin-releasing hormone receptor pathway	*MAP3K7*	Mitogen-activated protein kinase kinase kinase 7
Inflammation mediated by chemokine and cytokine signaling pathway	*MAP3K7*	Mitogen-activated protein kinase kinase kinase 7
Interleukin signaling pathway	*MAP3K7*	Mitogen-activated protein kinase kinase kinase 7
TGF-beta signaling pathway	*MAP3K7*	Mitogen-activated protein kinase kinase kinase 7
	TGFBR2	TGF-beta receptor type-2
Toll receptor signaling pathway	*MAP3K7*	Mitogen-activated protein kinase kinase kinase 7
Wnt signaling pathway	*MAP3K7*	Mitogen-activated protein kinase kinase kinase 7
p38 MAPK pathway	*MAP3K7*	Mitogen-activated protein kinase kinase kinase 7

Shaded box indicates immune-related signal pathway.

Supplementary Table S4. Signaling pathways of genes that interact with candidate genes of scrub typhus based on protein-protein interactions.

Signal Pathway	Gene	Count
5HT2 type receptor mediated signaling pathway	*PRKCQ*	1
ALP23B signaling pathway	*SMAD9*	1
Activin beta signaling pathway	*SMAD9*	1
Alzheimer disease-amyloid secretase pathway	*MAPK8, PAK1, PKN3, PKN2, MAPK9, PRKCQ, MAPK14*	7
Alzheimer disease-presenilin pathway	*CTNNB1, LEF1*	2

Angiogenesis	*PIK3R2, PIK3R1, MAPK8, MAP2K4, CTNNB1,FOS, PIK3R3, PIK3CB, STAT3, PDGFRA, JAK1, PRKCQ, JUN, PAK1, MAPK14*	15
Angiotensin II-stimulated signaling through G proteins and beta-arrestin	*ARRB2*	1
Apoptosis signaling pathway	*CHUK, DAXX, MAPK8,MAP2K4, HSPA1L, RIPK1, ATF4, ATF2, FOS, XIAP, JDP2, TRAF2, MAP4K1, TNFRSF1A, MAP4K4, MAP2K7, MAP3K14, MAPK9,PIK3CB, MAP3K5, PRKCQ, JUN, IKBKB, RELA*	24
Axon guidance mediated by netrin	*PIK3R2, PIK3R1, PIK3R3, PIK3CB*	4
Axon guidance mediated by semaphorins	*NRP1, PAK1*	2
B cell activation	*CHUK, MAPK8, MAP3K3, FOS, MAPK9, PIK3CB, JUN, IKBKB, MAPK14*	9
BMP/activin signaling pathway-drosophila	*SMAD9*	1
CCKR signaling map	*PIK3R1, MAPK8, MAP2K4, ARRB2, CTNNB1, ATF2, FOS, TRAF6, MAP2K6, MAP3K14, MAPK9, PIK3CB, STAT3, PRKCQ, JUN, PAK1, MAPK14*	17
Cadherin signaling pathway	*CELSR2, CTNNB1, ERBB2, LEF1*	4
Cell cycle	*CCNB1, PSMD11*	2
Cytoskeletal regulation by Rho GTPase	*PAK1*	1
DNA replication	*HIST2H3D, HIST2H3C*	2
DPP signaling pathway	*SMAD9*	1
Dopamine receptor mediated signaling pathway	*FLNA*	1
EGF receptor signaling pathway	*CBL,MAPK8, MAP2K4, MAP3K3, YWHAE, PPP6C, ERBB2, MAP2K6, MAP2K7, MAPK9, PIK3CB, MAP3K5, STAT3, PRKCQ, MAPK14*	15
Endothelin signaling pathway	*PIK3R2, PIK3R1, PIK3R3, PIK3CB, PRKCQ*	5
FAS signaling pathway	*DAXX, MAPK8, MAP2K4, MAPK9, MAP3K5, JUN*	6
FGF signaling pathway	*MAPK8, MAP2K4, MAP3K3, YWHAE, PPP6C, MAP2K6, MAP2K7, MAPK9, PIK3CB, MAP3K5, PRKCQ, MAPK14*	12
GBB signaling pathway	*SMAD9*	1
Glycolysis	*NLK*	1
Gonadotropin-releasing hormone receptor pathway	*BMPR1A, PIK3R1, ACVR2B, MAPK8, NR3C1, MAP3K3, KAT2B, CTNNB1, SMAD3, ATF2, TGFB2, FOS, MAP3K7, MAP2K6, MAP4K1, MAP4K4, MAP2K7, TGFB1, MAP3K14, SMAD9, EP300, CREBBP, MAPK9, JUND, MAP3K5, STAT3, PRKCQ, JUN, JUNB, RELA, MAPK14*	31
Hedgehog signaling pathway	*CREBBP*	1
Heterotrimeric G-protein signaling pathway-Gi alpha and Gs alpha mediated pathway	*ARRB2, EP300, CREBBP*	3
Heterotrimeric G-protein signaling pathway-Gq alpha and Go alpha mediated pathway	*PRKCQ*	1
Histamine H1 receptor mediated signaling pathway	*PRKCQ*	1
Huntington disease	*MAP2K4, FOS, MAP2K7, EP300, CREBBP, MAPK9, JUN*	7
Hypoxia response via HIF activation	*PIK3R2, PIK3R1, PIK3R3, CREBBP, PIK3CB*	5
Inflammation mediated by chemokine and cytokine signaling pathway	*CHUK, ARRB2, MAP3K7, PIK3CB, JUND, STAT3, JUN, JUNB, IKBKB, PAK1, RELA*	11

Insulin/IGF pathway-mitogen activated protein kinase kinase/MAP kinase cascade	*FOS*	1
Insulin/IGF pathway-protein kinase B signaling cascade	*PIK3R2, PIK3R1, PIK3R3, PIK3CB*	4
Integrin signaling pathway	*PIK3R2, PIK3R1, MAPK8, MAP2K4, MAP3K3, PIK3R3, MAPK9, PIK3CB, MAP3K5, FLNA*	10
Interferon-gamma signaling pathway	*MAPK8, MAPK9, JAK1, MAPK14*	4
Interleukin signaling pathway	*CHUK, FOS, MAP3K7, PIK3CB, STAT3, IKBKB*	6
JAK/STAT signaling pathway	*STAT3, JAK1, MAPK14*	3
MYO signaling pathway	*SMAD9*	1
Muscarinic acetylcholine receptor 1 and 3 signaling pathway	*PAK1, PKN3, PKN2, PRKCQ*	4
Nicotine pharmacodynamics pathway	*FLNA*	
Oxidative stress response	*MAPK8, MAP2K4, ATF2, MAP2K6, MAPK9, DDIT3, JUN, MAPK14*	8
Oxytocin receptor mediated signaling pathway	*PRKCQ*	1
PDGF signaling pathway	*CHUK, PIK3R2, PIK3R1, MAPK8, COPS5, ELP1, FOS, PIK3R3, PKN2, PIK3CB, STAT3, PDGFRA, JAK1, JUN, IKBKB*	15
PI3 kinase pathway	*PIK3R2, PIK3R1, PIK3R3, PIK3CB*	4
Parkinson disease	*MAPK8, YWHAE, HSPA1L, MAPK9, MAPK14*	5
Pentose phosphate pathway	*NLK*	1
Ras pathway	*MAPK8, MAP2K4, ATF2, MAP2K6, MAP2K7, MAPK9, PIK3CB, STAT3, JUN, PAK1, MAPK14*	11
SCW signaling pathway	*SMAD9*	
T cell activation	*CHUK, PIK3R2, PIK3R1, MAPK8, FOS, PIK3R3, MAPK9, PIK3CB, PRKCQ, JUN, IKBKB, PAK1*	12
TGF-beta signaling pathway	*SMAD6, BMPR1A, ACVR2B, MAPK8, SMURF1, TAB1, SMAD3, SMURF2, ATF2, TGFB2, MAP3K7, TGFBR1, ACVRL1, SMAD7, TGFB1, SMAD9, TGFB3, EP300, CREBBP, MAPK9, JUND, JUN, JUNB, MAPK14, TGFBR2*	25
Thyrotropin-releasing hormone receptor signaling pathway	*PRKCQ*	1
Toll receptor signaling pathway	*CHUK, MAPK8, TLR3, TAB1, MAP3K7, TRAF6, IRAK1, MAPK9, JUN, TRAF2, IKBKB, RELA, MAPK14*	13
Transcription regulation by bZIP transcription factor	*EP300, CREBBP*	2
Ubiquitin proteasome pathway	*SMURF1, SMURF2, PSMD11*	3
VEGF signaling pathway	*PIK3R2, PIK3R1, PIK3R3, PIK3CB, PRKCQ, MAPK14*	6
Wnt signaling pathway	*BMPR1A, CELSR2, ARRB2, CTNNB1, NLK, MAP3K7, TGFBR1, SMAD9, EP300, CREBBP, PRKCQ, LEF1*	12
p38 MAPK pathway	*MAP2K4, TAB1, TAB2, MAP3K7, TRAF6, MAP2K6, MAP3K5, MAPK14*	8
p53 pathway feedback loops 2	*PIK3R2, PIK3R1, CTNNB1, PIK3R3, PIK3CB, MAPK14*	6
p53 pathway	*PIK3R2, PIK3R1, KAT2B, TRAF2, PIK3R3, EP300, CREBBP, CDK1, PIK3CB, CCNB1*	10

Shaded boxes indicate the signaling pathways related to over 10 genes that interact with candidate genes of scrub typhus based on protein-protein interactions.
